# The implementation of a longitudinal POCUS curriculum for physicians working at rural outpatient clinics in Chiapas, Mexico

**DOI:** 10.1186/s13089-018-0101-8

**Published:** 2018-08-15

**Authors:** Annie Heffernan Rominger, Gerardo Antonio Aguilar Gomez, Patrick Elliott

**Affiliations:** 1 Department of Pediatrics, Division of Emergency Medicine, University of Louisville, 571 South Floyd Street, Suite 412, Louisville, KY 40202 USA; 2 Compañeros En Salud, Calle Primera Poniente Sur #25, 30370 Angel Albino Corzo (Jaltenango), Chiapas Mexico

**Keywords:** POCUS education, Global health education, Point-of-care ultrasound (POCUS), Educational curriculum

## Abstract

**Background:**

Medical care in resource limited settings is challenging, particularly with limited access to diagnostic and imaging studies. The most portable and cost effective diagnostic imaging in these areas is ultrasound. Ultrasound is a very teachable skill with a short, single intensive training course and hands-on teaching; however, there are limited data on a longitudinal point-of-care ultrasound (POCUS) curriculum in resource limited settings. The goal of this intervention is to develop an effective longitudinal POCUS curriculum for local physicians working in rural clinics in the state of Chiapas, Mexico, and evaluate its effectiveness on patient care.

**Methods:**

This is a 12-month longitudinal ultrasound educational curriculum for local supervising physicians working in rural clinics in Chiapas, Mexico. The 10 clinics are a collaboration of the Mexican government and Compañeros En Salud with limited access to any diagnostic imaging or laboratory studies. The investigators assisted in obtaining four portable ultrasound machines for use in the clinics. Next, they organized four point-of-care ultrasound (POCUS) teaching sessions over a year, each session focusing on several distinct concepts. The sessions included lectures and hands-on teaching with both healthy volunteers and with patients in the various communities. Over the 12 months, the POCUS were logged and the majority of images saved. The logs were analyzed to determine if POCUS affected the medical management of the patients. The primary investigator reviewed 35.2% of the total ultrasounds completed, which was 52.2% of the save images, for quality assurance and feedback.

**Results:**

Over the 12 months, there were 584 ultrasound studies documented. The most common study was a transabdominal obstetric examination (45.5%) followed by abdomen/pelvis (26.6%) and musculoskeletal (5.7%) and skin and soft tissue (5.7%). The use of POCUS changed the patient diagnosis after 194 scans (34%) and changed the clinical management for the patient encounter in 171 (30%) scans. In the 194 scans in which POCUS changed the diagnosis, the clinical management was changed, as a direct result of the scan results, in 152 (78.4%) of those patient encounters.

**Conclusion:**

A longitudinal POCUS educational curriculum is an effective way to equip local physicians in resource limited countries with a tool to improve their clinical management of patients.

## Background

Medical care in resource-limited settings can present multiple challenges to the timely and accurate clinical diagnoses of patients. Typically, there are limited diagnostic and imaging capabilities available to medical providers. The most accessible diagnostic imaging modalities for primary care and emergency services in underserved areas are radiographs and ultrasounds [1]. If they are not readily available, lengthy transportation for appropriate diagnostic studies can delay treatment and result in increased costs to an already marginalized population [1, 2]. X-ray machines require more space, are more expensive than ultrasound machines, and expose the patients and staff to radiation. Consequently, since 1985, the World Health Organization (WHO) has recommended ultrasound for developing countries because it is portable, inexpensive, non-invasive, safe, and provides immediate information [3, 4].

Becker et al. performed a systematic review of the literature surrounding portable ultrasound use in low and middle income countries. The review included 27 original research articles and 9 case reports, which universally demonstrated effective screening or diagnostic uses of ultrasound for various conditions [5]. Some of these articles included Point-of-Care Ultrasound (POCUS) and others included traditional ultrasound examinations. As a definition, POCUS is performed by the treating physician and is intended to answer a specific question [2]. Diagnoses for which POCUS is suitable should fulfill two criteria: (1) it must be relevant to treatment decision making and (2) it must be easily and accurately recognizable by physicians applying ultrasound without the necessity for extended ultrasound training [6]. Therefore, one key strategy to make a lasting change in resource limited areas is to teach local providers to use POCUS so they can provide a higher level of care to the local people.

Shah et al. conducted an educational intervention in Rwanda to create an effective ultrasound curriculum for the providers at two of the local hospitals [3]. The investigators conducted a 9 week course which included a total of 15 lecture hours and 15–30 scanning hours The clinicians kept logs of their ultrasound studies and indicated that their findings changed the initial patient management in 43% of cases [7]. The most common change in patient management was the decision to perform a surgical procedure following the results of the ultrasound [7]. Overall, the study concluded that ultrasound is a very teachable skill with an intensive training course and hands-on teaching [3].

Another study concluded that ultrasound in low resource settings can provide adequate imaging in screening for placenta previa, fetal malposition, multiple gestations, ectopic pregnancy, obstructed labor, pelvic outlet measurement, and limited fetal anatomy [8]. These are all conditions that would necessitate a cesarean section to protect the life and well-being of both the mother and the neonate. They found that motivated trainees and an intensive and highly abbreviated training course by qualified physicians resulted in the achievement of basic ultrasound skills in much shorter training periods [8]. They also concluded that long-term success of the ultrasound deployment in resource limited areas also requires continuing education [8].

Partners in Health (PIH) is a nonprofit organization that launched Compañeros En Salud (CES) in 2011, which is a group that works with 10 of the rural government clinics in the Sierra Madre region of Chiapas, one of the most marginalized regions of Mexico [9]. Ultrasound is the only imaging modality available to the clinics and the nearest hospital with plain radiography is anywhere from 2 to 6 h away depending on the specific location of the clinic. The local people are very limited financially and traveling many hours away for basic tests or unnecessary referrals can cause a large financial strain on the person and family. Basic POCUS knowledge and skills may greatly aid in the local management of many medical conditions, reduce the number of referrals for imaging, and expedite the care of patients who require transfer for a serious medical or surgical problem.

As described above, short-term ultrasound training programs have proven to deliver adequate knowledge and skills to novices [4]. Most of the previous studies on training programs include a short single and sometimes repeat course taught by an outside group to local providers. Knowledge and skills can deteriorate without continuing education and long term success of an ultrasound program requires it [8]. In addition, a recent article examining POCUS programs in Africa suggests prioritizing longitudinal and sustainable models for training and short courses without ongoing supervision or skill maintenance should be avoided [10]. To the authors’ knowledge, there is not an evaluation of the effectiveness of a longitudinal POCUS curriculum in a clinic based resource limited setting. The immediate goal of this program is to develop an effective longitudinal, continuing POCUS curriculum for local CES physicians working in the state of Chiapas, Mexico and determine its effect on patient care in the area.

## Methods

This is a longitudinal educational curriculum initiated by the organizers of CES. CES recruits motivated Mexican physicians who are entering a government-required social service year (*pasantes*) to staff each of the 10 rural clinics. The supervisors in the organization are physicians who have completed their social service requirement are responsible for the education of the *pasantes*, administrative responsibilities, arranging transfer of patients, and oversight of the clinics. There are 6–8 supervisors each year and each one oversees 1–3 clinics. The ultrasound curriculum focuses on the supervisors since they are retained in the organization for a longer periods of time, travel between the clinics that they oversee, and are responsible for teaching the *pasantes*.

The instructors for the educational sessions included a Pediatric Emergency Medicine (PEM) physician, an Emergency Medicine (EM) physician, a PEM fellow, and two upper level EM residents from the University of Louisville. An Obstetrician from Harvard University/Brigham and Women’s Hospital was the instructor for the session on obstetric ultrasound. Both faculty members from the University of Louisville have extensive bedside ultrasound experience and have taught ultrasound at major national and international conferences.

The course lasted 4 days and it recurred every 3–4 months with different topics at each course. All the material was presented in English because all of the participants were fluent in English. The first 1.5 days were spent in the main offices with lectures (4 h) and hands on training (5 h) on healthy volunteers. The class ratio was 6–10 learners per two instructors. Then, they split up into two groups, each led by one of the ultrasound instructors, and went to the rural community clinics to ultrasound patients with known relevant pathology for the next 2 days (approximately 6–7 h each day). Prior to initiation of the course work, a needs assessment was conducted with the local physicians to determine the most relevant topics and the regional pathologies that are commonly encountered. The curriculum was then adjusted accordingly. All topics were covered over the 12 total months of the curriculum (four sessions). The total lecture time over the four sessions was 16 h and total hands-on time with healthy volunteers was 20 h. In addition, the trainees also received a total of 8 days, 6–7 h per day of hands-on training and bedside POCUS training on patients with known relevant diagnoses over the entire course. This added another 48–56 h of hands-on training to the course. The intent of breaking the course up into multiple sessions was to avoid overwhelming the learners and giving them time to master a couple POCUS topics before moving to the next ones. Multiple trips also allowed the opportunity to review interesting cases, technique, and individual competency from the previous topics. As most supervisors remain in their positions for several years, they re-review the material annually.

The investigators helped CES obtain four refurbished Sonosite nanomaxx ultrasound machines, each with low frequency (phased array) and high frequency (linear) probes. The supervisors were responsible for the ultrasound machines and took turns taking the machines with them to the clinics which they oversaw. Each supervisor had a machine for 2 weeks at a time and brought it to the clinics he or she supervised. While there, they worked with the *pasante* to schedule ultrasounds at the clinic for patients in the community who would benefit from imaging. They could also ultrasound patients if needed as they came into be evaluated. This gave the supervisors the opportunity to show the *pasantes* some concepts and uses of POCUS. Occasionally there would be case when ultrasound was urgently needed in one of the clinics and the supervisors would communicate and coordinate a way to get the machine to that clinic for that specific patient.

The first teaching session was in September 2015 and focused on introduction to ultrasound, care and use of the machine, focused assessment with sonography in trauma (FAST), evaluation of the kidney and bladder, and basic obstetrics. The next session was in February 2016 and it included lung ultrasound, skin and soft tissue, and musculoskeletal. The third session was conducted at the end of April 2016 by an OB/GYN physician and was focused on obstetrics. The fourth session was July 2016 and it reviewed ultrasound basics and then focused on cardiovascular, advanced abdomen, and ocular. The presentations from each session were left with the participants for their reference and review. At the completion of the curriculum, the investigator continued with two trips per year as a review and had 3–4 web based series of cases with images to review and discuss to promote long term competency.

There was an ultrasound log with each machine which documents the type of ultrasound conducted, reason for the study, presumed diagnosis, and if the ultrasound findings confirmed or changed the presumed diagnosis for the patient. The information included the patient’s initials, age, gender, and date of study. Lastly, the log contained the initials of the physician performing the ultrasound and site of the study. By providing the type of ultrasounds, it allowed the investigators to track the number of the different studies that were conducted and where more or less time should be spent in the future curriculum. By tracking the number of ultrasounds for each supervisor, the investigators could provide additional resources to those who were not as comfortable using the machines. Every 1–2 months, the supervisors brought the ultrasound logs to the monthly educational conferences where they were compiled from all the clinics, copied, and sent back to the primary investigator for review. The primary investigator transferred the information into a spreadsheet and each study was given a de-identified number and the initials of the person who received the study removed.

De-identified images were loaded onto a Google drive folder for review and feedback. The primary investigator reviewed the images for technique, quality and findings. Feedback was provided at each of the sessions or sooner via email as necessary. The primary investigator was available to review any images urgently if requested for clarification. This educational intervention was reviewed by the University of Louisville Institutional Review Board and deemed to be exempt. In addition, the study was reviewed by CES research committee and received approval to conduct this educational intervention.

## Results

It took 6 weeks after the initial session to get the ultrasound logs to all the machines and have consistent recording of the examinations. Therefore, only 10.5 months (December 2015–October 2016) of data were recorded and analyzed. Over the 12 months after the initiation of the curriculum, there were 584 ultrasound studies documented. The most common study was a transabdominal obstetric examination (45.5%) followed by abdomen/pelvis (26.6%), cardiovascular (5.8%), musculoskeletal (5.7%) and skin and soft tissue (5.7%) (Table 1). The number of ultrasounds performed by each supervisor ranged from 27 to 162 (Table 2). There were two supervisors who started their position halfway through the educational curriculum and therefore have less scans.

**Table 1:** The distribution of the types of ultrasound recorded over the study period

Type of ultrasound	Number of ultrasounds (*n*)	Percentage (%)
Obstetric	266	45.5
Abdomen	155	26.6
Cardiac	34	5.8
Breast	34	5.8
Skin and soft tissue	33	5.7
Musculoskeletal	33	5.7
Chest/lungs	17	2.9
Ocular	9	1.5
Thyroid	3	0.5
Total	584	100.0

**Table 2:** Number of ultrasounds recorded by each supervisor

Physician	Number of ultrasounds (*n*)	Percentage (%)
1	162	27.7
2	109	18.7
3	83	14.2
4	66	11.3
5	58	9.9
6	43	7.4
7	36	6.2
8	27	4.6
Total	584	100.0

The use of POCUS changed the diagnosis after 194 scans (34%) and changed the clinical management for the patient encounter in 171 (30%) scans. In the 194 scans in which POCUS changed the diagnosis, the clinical management was changed in 152 (78.4%) of those patient encounters. 113 of the included scans were completed under the direct supervision and feedback of one of the instructors during an educational session, leaving 471 scans completed independently. Of the scans completed without direct supervision, the use of POCUS changed the diagnosis after 33.7% of scans and changed the clinical management for the patient encounter in 30.7% of the scans.

Ocular ultrasound changed the diagnosis during the patient encounter most often with 62.5%, followed by skin and soft tissue (45.5%), cardiac (48%), and abdomen/pelvis (43.3%) (Table 3). POCUS changed the clinical management of the patient encounter the most after scans of the thyroid and eyes (both 50%), followed by cardiovascular (43.8%), musculoskeletal (42.4%), abdomen/pelvis (38%), breast (36.4%), and chest/lung (35.3%) (Table 4). The obstetric ultrasounds were the most commonly done and changed the diagnosis in 66 (24.4%) patient encounters and changed the management in 55 (20.2%) patient encounters.

**Table 3:** The frequency for which POCUS changed patient diagnosis in each of the different types of ultrasound examinations

Type of ultrasound	Total studies with data input	Numbers of changed diagnosis	Percent changed diagnosis
Obstetric	262	64	24.4
Abdomen/pelvis	150	65	43.3
Skin and soft tissue	33	15	45.5
Musculoskeletal	33	13	39.4
Breast	33	11	33.3
Cardiovascular	32	15	46.8
Chest/lungs	17	5	29.4
Ocular	8	5	62.5
Thyroid	2	1	50
Missing	15		
Total	584	194	33.2

**Table 4:** The frequency for which POCUS changed patient clinical management in each of the different types of ultrasound examinations

Type of ultrasound	Total studies with data input	Numbers of changed clinical management	Percentage of changed clinical management
OB	262	53	20.2
Abdomen/pelvis	150	57	38
Skin and soft tissue	33	10	30.3
Musculoskeletal	33	14	42.4
Breast	33	12	36.4
Cardiovascular	32	14	43.8
Chest/lungs	17	6	35.3
Ocular	8	4	50
Thyroid	2	1	50
Missing	14		
Total	584	171	29.2

**Fig. 1:**
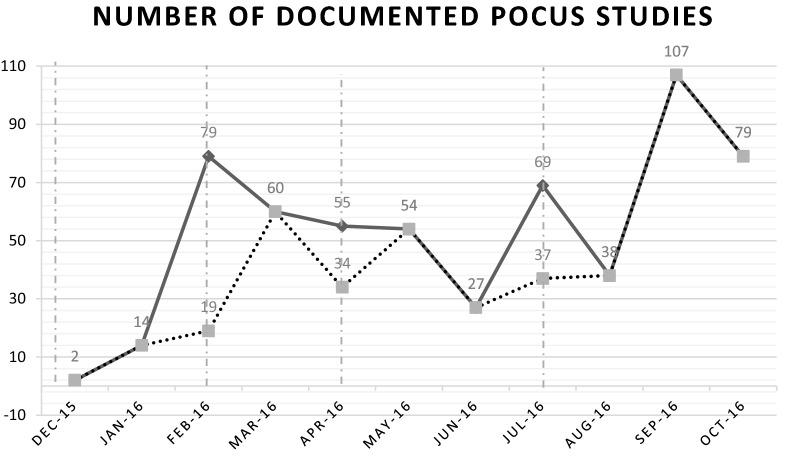
Linear representation of the total number of documented POCUS studies over the study period. The vertical dashed lines are the dates of the training sessions. The dotted line is the number of unsupervised documented POCUS studies over the study period

**Fig. 2:**
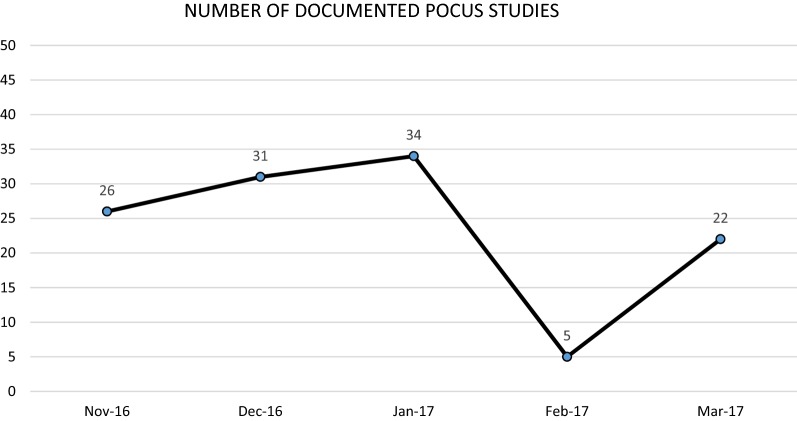
Linear representation of the total number of documented POCUS studies over the 5 months following the study period

The number of POCUS conducted per month typically spiked after a teaching session with gradual decrease in use over the following months (Fig. 1). Part of the spike was due to the scans completed during the sessions themselves (Fig. 1). The most scans were recorded in September 2016 which is 12 months after the initiation of the educational curriculum and after four educational sessions. In the 5 months after the completion of the educational curriculum, there were 128 scans completed (Fig. 2). There was a drop in scans in February when there was a change in supervisors. It is unclear whether the new supervisors were not logging the ultrasounds, they were not conducting as many ultrasounds, or the data was not entered. Despite this, the group still averaged > 25 scans per month. The most common imaging modalities remained to be obstetrics (56.3%) and abdomen/pelvis (25.1%). It changed diagnosis in 33.6% and changed management in 25.8% of the scans completed over the 5 month period.

There were a total of 584 included scans. 113 of the included scans were completed under the direct supervision and feedback of one of the instructors during an educational session, leaving 471 scans completed independently. There were 190 scans which were documented in the ultrasound log but no images were saved for review. Therefore, there were 281 scans that were not obtained under direct supervision of an instructor and had saved images for review. 33% of these scans were randomly selected and reviewed by the primary investigator to confirm the diagnosis and to provide feedback regarding technique. One of the instructors was either present or the primary investigator reviewed the scanned images for 206 of the 394 saved images (52.2%), which is 35.2% of the total 584 scans. There was a disagreement in findings in 4.3% of the images reviewed and none of which affected the clinical management of the patients. There were also 6.5% of scans with inadequate image quality to interpret.

## Discussion

The ultrasound use by local physicians following this longitudinal curriculum echoes the conclusions from previous studies that ultrasound is a very teachable and useful skill, particularly in resource limited areas. The previous study by Shah in Rwanda, showed increase ultrasound use 11 weeks after the departure of the ultrasound instructor following the ultrasound course with a high concordance rate of scan interpretation [7]. In US ED residents and faculty, there are improvements of ultrasound knowledge scores 6 months after training suggesting retention of the information [11]. Scores, however, were higher in those who received more hands-on training [11]. The total lecture time in this educational program was similar to previous single, short term educational programs; however, there was almost three times as much hands-on training, which has been shown to result in higher long term skill retention [7, 11]. Similar to the previous findings, the results show that ultrasound use peaks after an educational session; however they decrease to a plateau. After each session’s peak, the plateau was higher and higher suggesting more comfort with use following repeated sessions. The highest POCUS use in this study was 12 months after the initial course. Typically, ultrasound use wanes with time but repeated sessions that build on the previous clearly demonstrate an effective strategy for continued POCUS use and clinical application in resource limited settings. In addition, repeated sessions with skill reinforcement and technical review can serve as a revalidation of skills taught in a previous session(s) which is evidenced by a low disagreement rate of scan interpretation [10].

This study demonstrates how training and use of POCUS significantly affects the clinical management and diagnosis of patients in the marginalized region of the Sierra Madre in the state of Chiapas, Mexico. The 584 total scans recorded is a larger sample size than other similar studies since the population was followed for over 12 months. The 34% of patient encounters in which POCUS changed the clinical diagnosis likely prevented delays in care and expedited referral and hospitalization as needed without having to wait for a patient to obtain imaging at another site. In this study, ultrasound changed the management plan in 30% of patients. Although this is lower than the findings in Rwanda, which was a hospital based study with higher acuity patients, it shows the significant effect of POCUS in the resource limited primary care clinic based setting. As reported in previous ultrasound studies on inpatients, the remainder of the patients for which POCUS did not change diagnosis or management still benefit from a scan that can confirm a clinical diagnosis and reinforce the current management plan [2].

Similar to previous ultrasound studies in resource limited settings, the most commonly conducted ultrasound was obstetric. The investigators predicted this based on the initial needs assessment so obstetric ultrasound was taught at two educational sessions, including one by an obstetrician. Harris et al. showed that bedside ultrasound in resource limited areas, specifically Nicaragua and Africa is an effective way to identify serious obstetric conditions that can potentially save the life of the woman or the neonate [8]. Over the 12 month study period, some of obstetric POCUS findings that changed the diagnosis include placenta previa, twin pregnancy, molar pregnancy, incomplete spontaneous abortion, subchorionic hemorrhage, abnormal presentation (breech) of the fetus. Although the percent of obstetric scans for which there was change diagnosis is not extremely high, the diagnoses that were detected can be serious and potentially life threatening to the woman or the fetus/neonate in resource limited areas. Maternal and child health is a major health initiative in the developing world and different cultural barriers and traditions present challenges to physicians in these areas [8]. Many women in the region of Chiapas and other resource limited settings have out of hospital deliveries, typically with a traditional birth attendant. By providing the physicians with ultrasounds and appropriate training, they can identify women who are at increased risk of complications and make earlier referral for hospitalized delivery or cesarean section.

## Limitations

The major limitation of the analysis of the educational curriculum is an incomplete ultrasound log. The supervisors may not have remembered to record every scan conducted and of those recorded, there were 190 which were not saved. Although this could affect the descriptive data, it would result in the underestimation of the use of ultrasound in the clinics. Since the goal of the curriculum is increase in POCUS use, it does not have an overall impact on the intervention. However, it may have contributed to the primary investigators review of the scans for quality assurance. Another limitation is supervisor turnover during the educational curriculum. There were two supervisors who left for other positions after the first session and two new supervisors were added after the 2nd session. This may have skewed the results since it was not a consistent population of learners throughout the entire curriculum. The two most commonly performed studies were taught during the first session which may have contributed to the large number of these studies and skewed the results.

The investigators did not collect a written or hands-on examination of the course participants to evaluate their improvement in knowledge and skill retention. Although their knowledge would have increased since they reported not having little to no knowledge and experience with ultrasound, having an objective assessment of their knowledge would have been helpful. In addition, a hands-on session would have been helpful to evaluate skill retention. This is another limitation of the evaluation of the educational curriculum. Lastly, although the local physicians were fluent in English, the course was not given in their native language so there may have been comprehension issues that were not apparent during the course.

## Conclusion

A longitudinal POCUS educational curriculum is an effective means to teach ultrasound in resource limited settings. It can equip local physicians with a tool to guide their clinical diagnoses and improve their clinical management of patients. In addition, the POCUS program conducted in rural Chiapas, Mexico demonstrates its usefulness in the resource limited outpatient clinic setting.

## Abbreviations

POCUS: Point-of-care ultrasound
CES: Compañeros En Salud
PIH: Partners in Health
EM: Emergency Medicine
